# Longitudinal relationships between physical exercise, psychological resilience, and academic engagement in Chinese university students: a random-intercept cross-lagged panel study

**DOI:** 10.3389/fpsyg.2026.1989887

**Published:** 2026-10-13

**Authors:** Yuankai Qiao, Haibin Liu

**Affiliations:** School of Physical Education, Liaoning Normal University, Dalian, Liaoning, China

**Keywords:** academic engagement, cross-lagged panel model, longitudinal study, physical exercise, psychological resilience, random-intercept cross-lagged panel model, university students

## Abstract

The relationships among physical exercise, psychological resilience, and academic engagement have predominantly been examined using cross-sectional designs, which preclude inference about directionality. This study examined their longitudinal relationships in a two-wave panel of Chinese university students. Of 1,024 students who provided valid baseline data (October 2024), 876 (Mage = 20.34, SD = 1.67; 58.3% female) provided valid follow-up data 6 months later (April 2025) on exercise volume (PARS-3), psychological resilience (CD-RISC-10), and academic engagement (UWES-S). Cross-lagged panel models (CLPM) and random-intercept cross-lagged panel models (RI-CLPM) were estimated in Mplus 8.8, with longitudinal measurement invariance established for all instruments. In the CLPM, exercise volume at T1 predicted resilience at T2 (beta = 0.17, *p* < 0.001) and resilience predicted engagement at T2 (beta = 0.14, *p* = 0.003), whereas the reverse paths and the direct exercise-to-engagement path were not significant. In the RI-CLPM, which separates between-person from within-person variance, the within-person association of exercise volume with subsequent resilience remained significant (beta = 0.11, *p* = 0.009) and was unaffected by assumptions about the reliability of the single-indicator exercise measure, whereas the within-person resilience-to-engagement path was attenuated (beta = 0.07, *p* = 0.074). Neither gender nor age moderated the relationships. Exercise volume is thus prospectively associated with subsequent resilience at the within-person level, whereas the resilience-engagement link appears to operate primarily between persons. Because the two-wave design and the single-indicator exercise measure require identifying constraints, the within-person decomposition is model-implied, and these findings are best interpreted as temporally ordered associations rather than confirmed within-person causal effects; exercise promotion stands as a candidate strategy for resilience support in university populations, pending experimental confirmation.

## 1 Introduction

Academic life at university concentrates a distinctive set of pressures into a short developmental window. Course load, continuous assessment, competition for scholarships and graduate placements, and the approach of labor-market entry all peak at the same time, and survey evidence using general-population benchmarks shows substantially higher psychological distress among university students than among age-matched peers (Stallman, 2010). A synthesis of evidence from secondary and higher education links sustained academic stress to impaired sleep, lowered wellbeing, and weakened academic performance, and identifies university students as a group for whom stress-management resources are often scarce (Pascoe et al., 2020). In China these pressures take a specific form: entry into higher education releases students from the tightly supervised routines of secondary schooling at precisely the moment when self-directed study becomes decisive, so the habits formed in the first undergraduate years carry long consequences (Auerbach et al., 2018; Kuh, 2009). Sustained engagement with study is among the clearest protective outcomes in this setting, because it forecasts persistence, performance, and timely graduation (Salmela-Aro and Upadyaya, 2014). Identifying modifiable behaviors that protect students’ capacity to stay engaged under pressure is therefore a practical priority, and two candidates stand out: physical exercise and psychological resilience.

Physical exercise is among the most consistently supported of these behaviors. In a sample of 1.2 million United States adults, exercisers reported markedly fewer days of poor mental health than matched non-exercisers (Chekroud et al., 2018), and systematic reviews of younger populations converge on similar associations (Rodriguez-Ayllon et al., 2019). Part of the explanation may run through psychological resilience, the capacity to adapt successfully to adversity (Connor and Davidson, 2003). Developmental research treats resilience as arising from ordinary, trainable processes rather than rare personal gifts (Masten, 2001), and the modal response to adversity is a stable trajectory of healthy functioning rather than breakdown (Bonanno, 2004). Exercise supplies several of the experiences those ordinary processes feed on: repeated exposure to manageable physical challenge can act as stress inoculation, fitness training recalibrates stress-regulatory physiology (Silverman and Deuster, 2014), and the positive affect generated during activity builds durable personal resources over time (Fredrickson, 2001; Tugade and Fredrickson, 2004). The exercise-resilience pairing is thus grounded in mechanism as well as in correlation.

Whether resilience then translates into academic engagement is a separate question. The Conservation of Resources (COR) theory holds that individuals strive to obtain, retain, and protect valued resources, and that initial resource gains beget further gains in resource spirals (Hobfoll, 2002). COR further distinguishes resources by their stability: some accumulate over long periods into trait-like resource caravans, whereas others fluctuate as shorter-lived states (Hobfoll et al., 2018). Physical exercise can be read as a resource-building activity, resilience as the personal resource it builds, and academic engagement as the behavioral investment that resilient students are better placed to sustain during demanding periods, in line with demands-resources accounts of study engagement (Bakker and Demerouti, 2017).

The direct exercise-engagement link deserves its own analysis, because competing intuitions point in opposite directions. On one side, research on physical activity and cognition documents better executive function and attention among habitually active young people, and school-based studies associate sports participation with stronger achievement once family background is taken into account (Hillman et al., 2008; Trudeau and Shephard, 2008; Donnelly et al., 2016). On the other side, a widely held belief among Chinese students and their parents holds that hours spent on the sports field displace hours spent at the desk, so that exercise drains rather than feeds academic effort. Both views are plausible, and neither has been tested against within-person longitudinal data in this population, which is one reason the present study treats the direct path as an open empirical question.

Almost all of this evidence is cross-sectional, and cross-sectional correlations cannot establish temporal precedence or exclude reverse causality: resilient students may simply be more inclined to exercise, and engaged students may accumulate mastery experiences that feed back into resilience. The cross-lagged panel model (CLPM) has served as the standard longitudinal tool for such questions, but it conflates stable between-person differences with time-varying within-person processes, and both simulation and empirical work show that its lagged coefficients can mislead when the focal process is genuinely within-person (Hamaker et al., 2015; Berry and Willoughby, 2017; Lucas, 2023). The random-intercept cross-lagged panel model (RI-CLPM) adds person-specific intercepts that absorb trait-like stability, so that the remaining paths describe what happens when a student deviates from their own baseline (Hamaker et al., 2015; Mulder and Hamaker, 2021; Orth et al., 2021).

Chinese university students are a particularly relevant population for this pair of models. Habitual physical activity declines sharply after secondary school in China at the same time as academic and social pressures intensify, and longitudinal tests that separate between-person from within-person variance in this group remain scarce. If exercise volume is prospectively associated with resilience at the within-person level, exercise promotion becomes a candidate component of university mental-health strategy rather than remaining a fitness initiative.

The present study followed four aims. First, we estimated a CLPM to test the temporal ordering of the three constructs. We predicted that students reporting more exercise at baseline would report higher resilience six months later (Hypothesis 1), that more resilient students would report stronger subsequent engagement (Hypothesis 2), and that the direct path from exercise to engagement would be small and positive rather than negative, contrary to the time-displacement belief (Hypothesis 3). The reverse paths were examined as an explicit research question (Research Question 1), because selection effects in both directions are plausible. Second, we re-estimated the model as an RI-CLPM, predicting that the exercise-to-resilience path would survive the separation of between-person variance (Hypothesis 4) while the resilience-to-engagement path would prove largely between-person in nature (Hypothesis 5). Third, we established longitudinal measurement invariance for all instruments. Fourth, we tested gender and age as moderators.

## 2 Methods

### 2.1 Participants and procedure

Participants were undergraduate students recruited from three public universities in Dalian, Liaoning Province, China, using convenience sampling. The baseline survey (T1) was administered in October 2024 through the Wenjuanxing online platform during regular class hours; 1,062 students submitted responses, of whom 38 failed the embedded attention checks, leaving 1,024 valid baseline records. The follow-up survey (T2), administered 6 months later in April 2025 using the same procedure, was linked to T1 responses through pseudonymized participant codes: each student held a numeric identifier assigned by class administrators, entered it at both waves, and the code list was stored separately from all survey responses. Of the 1,024 baseline respondents, 897 completed T2, and 21 of these failed the T2 attention checks, leaving a final analytic sample of 876 students (an overall loss of 14.5% of valid baseline records; Mage = 20.34, SD = 1.67; 58.3% female, 41.7% male) spanning all four undergraduate years; 11.8% were physical education or sport-science majors. Each survey took approximately 15 min to complete. Inclusion criteria were full-time undergraduate enrollment at one of the participating universities and provision of informed consent; exclusion criteria were failure of an attention check at either wave or non-completion of the follow-up survey. There were no exclusions based on major or health condition, because the study aimed to represent the general student population rather than a sport-active subgroup. Attrition analyses indicated no significant differences between completers and non-completers on any T1 study variable (all *p* > 0.05; standardized mean differences below 0.10; Table 1). All participants provided written informed consent before the baseline survey, and the study protocol was approved by the Ethics Committee of Liaoning Normal University (approval number LNNU-2024-PE-015).

**Table 1:** Attrition analysis: completers versus non-completers on baseline variables.

Variable	Completers (*n* = 876) M (SD)	Non-completers (*n* = 148) M (SD)	*t*/chi-square	*p*	SMD
T1 exercise volume	35.17 (18.24)	34.52 (19.06)	*t* = 0.41	0.682	0.03
T1 resilience	26.93 (6.32)	26.41 (6.55)	*t* = 0.93	0.354	0.08
T1 academic engagement	37.46 (8.51)	36.88 (8.93)	*t* = 0.74	0.458	0.07
Age (years)	20.34 (1.67)	20.48 (1.70)	*t* = −0.97	0.334	0.08
Female (%)	58.3	55.4	chi-square = 0.42	0.517	0.06

Non-completers comprise baseline respondents who either did not complete the follow-up survey or failed its attention checks. SMD, standardized mean difference (Cohen’s *d* for continuous variables, difference in proportions for gender). T1, Time 1.

### 2.2 Measures

Physical exercise was assessed with the Physical Activity Rating Scale-3 (PARS-3; Liang, 1994), a Chinese instrument that evaluates typical exercise participation over the preceding month on three rated dimensions: intensity, duration, and frequency, each scored from 1 to 5. The intensity anchors run from very light to very strenuous effort, the duration anchors from 10 to 20 min per session to more than 120 min, and the frequency anchors from less than once per month to every day. Following the standard scoring algorithm, the total exercise volume score is computed as intensity multiplied by (duration minus one) multiplied by frequency, yielding a range from 0 to 100, with higher scores indicating greater exercise volume; scores of 19 or below, 20 to 42, and 43 or above conventionally denote low, moderate, and high exercise levels. Because the total is an algorithmic composite rather than a sum of interchangeable reflective items, internal-consistency coefficients are not meaningful for this scale; its 6-month rank-order stability (0.63) is reported instead. In the present sample the mean volume (35.17 at T1) fell in the moderate band.

Psychological resilience was measured with the 10-item Connor-Davidson Resilience Scale (CD-RISC-10; Campbell-Sills and Stein, 2007), a unidimensional measure in which items are rated on a 5-point scale from 0 (not true at all) to 4 (true nearly all the time), with total scores ranging from 0 to 40. Representative items include “I am able to adapt when changes occur” and “I tend to bounce back after illness or hardship.” The Chinese adaptation used here has demonstrated sound psychometric properties (Wang et al., 2010). Cronbach’s alpha was 0.90 at T1 and 0.91 at T2.

Academic engagement was measured with the 9-item student version of the Utrecht Work Engagement Scale (UWES-S; Schaufeli et al., 2002), which comprises three 3-item facets: vigor (e.g., “I feel strong and vigorous when I study”), dedication (e.g., “I am enthusiastic about my studies”), and absorption (e.g., “I am immersed in my studies”). Items are rated on a 7-point frequency scale from 0 (never) to 6 (always), and total scores range from 0 to 54, with higher total scores indicating stronger engagement. Cronbach’s alpha was 0.88 at T1 and 0.89 at T2.

### 2.3 Analytical strategy

Analyses were conducted in Mplus 8.8 (Muthén and Muthén, 2017) with robust maximum likelihood (MLR) estimation to accommodate departures from multivariate normality, and the small amount of item-level missing data was handled with full-information maximum likelihood. Longitudinal measurement invariance was tested for each multi-item instrument through a hierarchical sequence of configural, metric, and scalar models; invariance was considered tenable when the decrease in CFI did not exceed 0.010 and the increase in RMSEA did not exceed 0.015 between nested models (Chen, 2007). The full invariance sequence for each instrument is reported in Table 2. The traditional CLPM was then specified with each T2 variable regressed on all three T1 variables, with T1 variables freely correlated and concurrent T2 residuals allowed to covary. The RI-CLPM added a random intercept for each construct, with loadings fixed at 1 on both occasions, so that the structural paths among the within-person components estimate within-person dynamics (Hamaker et al., 2015). Because the PARS-3 is a single observed indicator, its random-intercept variance, within-person residual variance, and measurement error are not jointly identified without constraints; we therefore constrained the within-person component variances of exercise to equality across waves and, in sensitivity analyses, fixed the indicator error variance to values implied by reliabilities of 0.70, 0.80, and 0.90, with the error-free specification reported as the main model. The variance shares reported below should accordingly be read as model-implied quantities under these constraints rather than as directly estimable ones. Under these constraints, the RI-CLPM paths describe temporally ordered within-person associations that are conditional on the assumed reliability structure; they cannot establish causal within-person effects, and two measurement occasions cannot identify individual developmental trajectories, both of which would require three or more waves or experimental manipulation (Maxwell and Cole, 2007; Mulder and Hamaker, 2021). The RI-CLPM is therefore reported as a complement to the CLPM that separates stable between-person differences from time-specific fluctuations under explicitly stated assumptions, not as definitive evidence of within-person causal processes. Model fit was evaluated with CFI and TLI (values of 0.95 or higher indicating good fit), RMSEA (0.06 or lower), and SRMR (0.08 or lower), and the two models were compared with the Satorra-Bentler scaled chi-square difference test. Power for the focal within-person path was examined through a Monte Carlo simulation with 1,000 replications using the final parameter estimates as population values (Muthén and Muthén, 2002); estimated power was 0.83 for the within-person exercise-to-resilience path and above 0.95 for the CLPM cross-lagged paths. Indirect effects were estimated with 5,000 bias-corrected bootstrap draws; because mediator and outcome were both measured at T2 and two waves cannot identify a longitudinal mediation chain, these estimates are reported as exploratory and descriptive rather than as formal mediation tests (Maxwell and Cole, 2007). Moderation by gender was examined with multi-group likelihood ratio tests comparing models with cross-lagged paths constrained versus freely estimated across groups, and moderation by age was tested with latent interaction terms. All tests were two-tailed with alpha set at 0.05. R-squared values for the endogenous variables are reported in Table 3.

**Table 2:** Longitudinal measurement invariance for the multi-item instruments.

Instrument	Model	chi-square	df	CFI	TLI	RMSEA	SRMR	Delta CFI	Delta RMSEA
CD-RISC-10	Configural	78.15	64	0.971	0.964	0.048	0.040	—	—
	Metric	91.40	71	0.968	0.962	0.049	0.042	−0.003	0.001
	Scalar	108.26	78	0.963	0.957	0.051	0.044	−0.005	0.002
UWES-S	Configural	96.44	74	0.958	0.949	0.052	0.043	—	—
	Metric	112.07	81	0.953	0.946	0.053	0.045	−0.005	0.001
	Scalar	131.35	88	0.946	0.939	0.057	0.048	−0.007	0.004

*N* = 876. Robust maximum likelihood estimation. Delta values refer to the comparison with the preceding, less constrained model. The PARS-3 was modeled as a single observed indicator; no invariance sequence applies.

**Table 3:** Standardized parameter estimates for the CLPM and RI-CLPM (*N* = 876).

Parameter	CLPM beta	95% CI	*p*	RI-CLPM beta	95% CI	*p*
Autoregressive: exercise	0.47	[0.41, 0.53]	<0.001	0.27	[0.08, 0.46]	0.005
Autoregressive: resilience	0.43	[0.37, 0.49]	<0.001	0.22	[0.03, 0.41]	0.027
Autoregressive: engagement	0.36	[0.30, 0.42]	<0.001	0.16	[0.01, 0.31]	0.041
Exercise (T1) → Resilience (T2)	0.17	[0.09, 0.25]	<0.001	0.11	[0.02, 0.20]	0.009
Resilience (T1) → Engagement (T2)	0.14	[0.06, 0.22]	0.003	0.07	[−0.01, 0.15]	0.074
Exercise (T1) → Engagement (T2)	0.05	[−0.01, 0.11]	0.128	0.03	[−0.04, 0.10]	0.312
Resilience (T1) → Exercise (T2)	0.06	[−0.03, 0.15]	0.178	0.04	[−0.03, 0.11]	0.265
Engagement (T1) → Resilience (T2)	0.03	[−0.05, 0.11]	0.462	0.02	[−0.05, 0.09]	0.583
Indirect: exercise → resilience → engagement (exploratory)	0.03	[0.01, 0.05]	0.011	—	—	—
R-squared: T2 exercise	0.23			0.08 (within)		
R-squared: T2 resilience	0.30			0.07 (within)		
R-squared: T2 engagement	0.25			0.03 (within)		

RI-CLPM cross-lagged and autoregressive paths are within-person estimates. R-squared values in the RI-CLPM refer to the within-person components of the T2 variables. The indirect effect is reported as an exploratory descriptive estimate (mediator and outcome both at T2).

## 3 Results

### 3.1 Descriptive statistics and correlations

Participant flow from recruitment to the analytic sample is summarized in Figure 1, and the attrition analysis appears in Table 1. Table 4 presents the means, standard deviations, and correlations among the study variables. All three variables showed moderate to high rank-order stability across the 6-month interval, with test–retest correlations of 0.63 for exercise, 0.57 for resilience, and 0.55 for academic engagement (all *p* < 0.001). At T1, exercise correlated positively with resilience (*r* = 0.27, *p* < 0.001) and with engagement (*r* = 0.19, *p* < 0.001), and resilience and engagement were substantially intercorrelated (*r* = 0.44, *p* < 0.001). The cross-time correlations showed the same general pattern: T1 exercise correlated with T2 resilience (*r* = 0.23, *p* < 0.001) and T2 engagement (*r* = 0.15, *p* < 0.001), and T1 resilience correlated with T2 engagement (*r* = 0.34, *p* < 0.001). These zero-order associations are consistent with, but do not by themselves establish, the hypothesized directional pathways.

**Figure 1:**
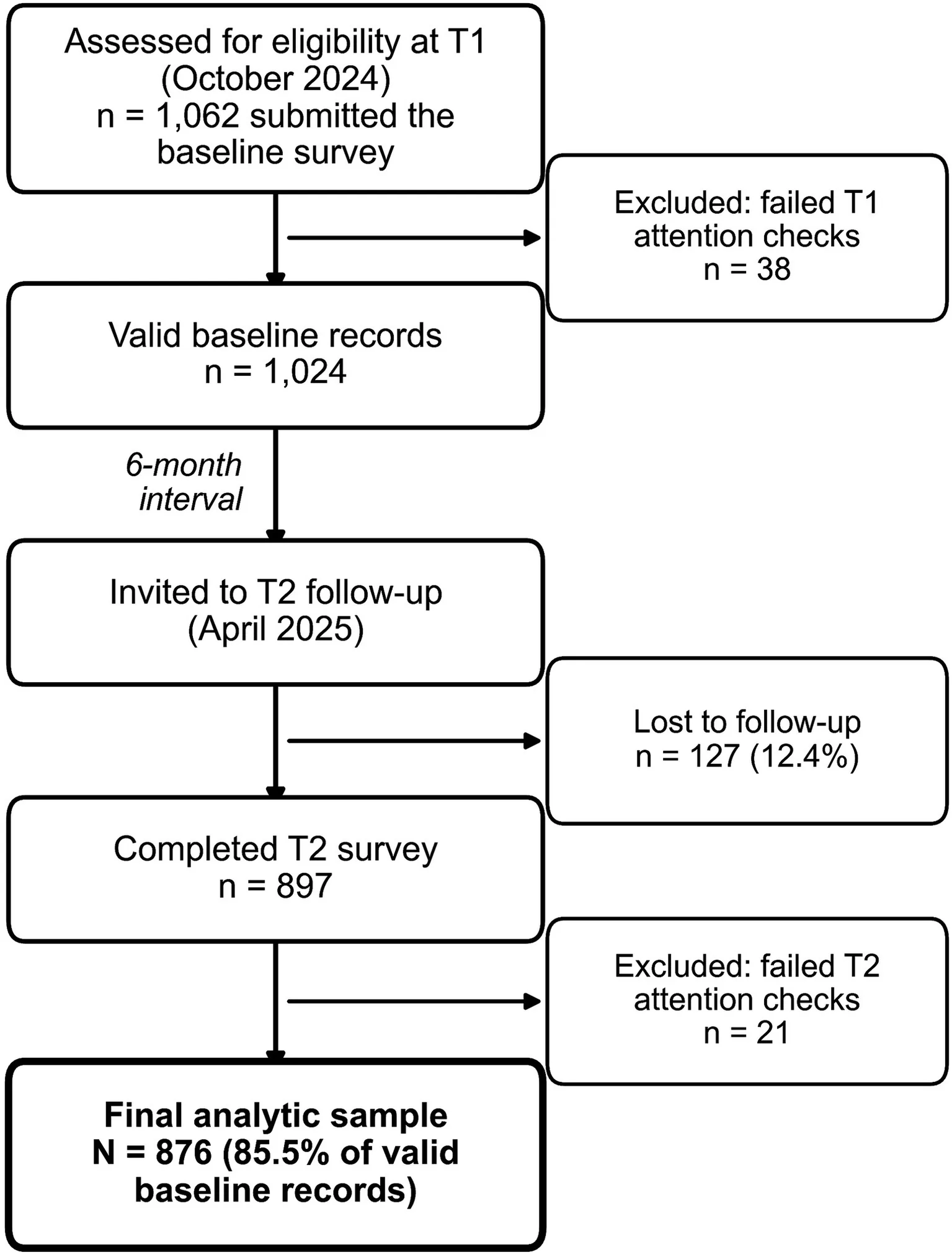
Participant flow from recruitment to the analytic sample. Of 1,062 students who submitted the baseline survey, 38 failed the embedded attention checks, leaving 1,024 valid baseline records. At the 6-month follow-up, 897 students completed the survey and 21 of these failed the attention checks, leaving a final analytic sample of 876. Percentages based on valid baseline records (N = 1,024).

**Table 4:** Descriptive statistics and correlations among study variables (*N* = 876).

Variable	M (SD)	1	2	3	4	5
1. T1 Exercise	35.17 (18.24)	—	0.27**	0.19**	0.63**	0.23**
2. T1 Resilience	26.93 (6.32)		—	0.44**	0.16**	0.57**
3. T1 Engagement	37.46 (8.51)			—	0.14**	0.32**
4. T2 Exercise	34.28 (18.87)				—	0.25**
5. T2 Resilience	27.31 (6.64)					—
6. T2 Engagement	36.72 (8.43)	0.15**	0.34**	0.55**	0.18**	0.43**

T1, Time 1; T2, Time 2 (6-month follow-up). **p < 0.01.

### 3.2 Measurement invariance

Longitudinal measurement invariance was supported for all multi-item instruments. The full sequence of configural, metric, and scalar models for each instrument is reported in Table 2, with chi-square, df, CFI, TLI, RMSEA, SRMR, and the changes in CFI and RMSEA at each step. For the CD-RISC-10, the scalar model fit acceptably (CFI = 0.963, TLI = 0.957, RMSEA = 0.051, SRMR = 0.044; delta CFI = −0.005, delta RMSEA = 0.002 relative to the metric model). For the UWES-S, scalar invariance was also supported (CFI = 0.946, TLI = 0.939, RMSEA = 0.057, SRMR = 0.048; delta CFI = −0.007, delta RMSEA = 0.004 relative to the metric model). Because the PARS-3 uses an algorithmic composite rather than a reflective item set, it was modeled as a single observed indicator and no invariance sequence applies. These results justify comparing latent structural parameters across the two waves.

### 3.3 Cross-lagged panel model

The CLPM fit the data well (chi-square = 189.62, df = 112, *p* < 0.001; CFI = 0.971, TLI = 0.963, RMSEA = 0.043, 90% CI [0.032, 0.053], SRMR = 0.040), and full parameter estimates are presented alongside the RI-CLPM estimates in Table 3 and Figure 2. All autoregressive paths were significant: exercise (beta = 0.47, 95% CI [0.41, 0.53], *p* < 0.001), resilience (beta = 0.43, 95% CI [0.37, 0.49], *p* < 0.001), and engagement (beta = 0.36, 95% CI [0.30, 0.42], *p* < 0.001), indicating moderate stability over the interval. Consistent with Hypothesis 1, the cross-lagged path from T1 exercise to T2 resilience was significant (beta = 0.17, 95% CI [0.09, 0.25], *p* < 0.001), and consistent with Hypothesis 2, the path from T1 resilience to T2 engagement was also significant (beta = 0.14, 95% CI [0.06, 0.22], *p* = 0.003). Hypothesis 3 received no clear support: the direct path from T1 exercise to T2 engagement was small and not significant (beta = 0.05, 95% CI [−0.01, 0.11], *p* = 0.128), although its direction was positive rather than negative, so the data offer no support for the time-displacement view. Neither reverse path approached significance: T1 resilience did not predict T2 exercise (beta = 0.06, *p* = 0.178), and T1 engagement did not predict T2 resilience (beta = 0.03, *p* = 0.462). The model explained 23%, 30%, and 25% of the variance in T2 exercise, resilience, and engagement, respectively. As an exploratory descriptive estimate, the indirect effect from T1 exercise to T2 engagement through T2 resilience was small (indirect beta = 0.03, 95% CI [0.01, 0.05], *p* = 0.011); given that mediator and outcome were both measured at T2, this quantity should not be read as a longitudinal mediation test (Maxwell and Cole, 2007).

**Figure 2:**
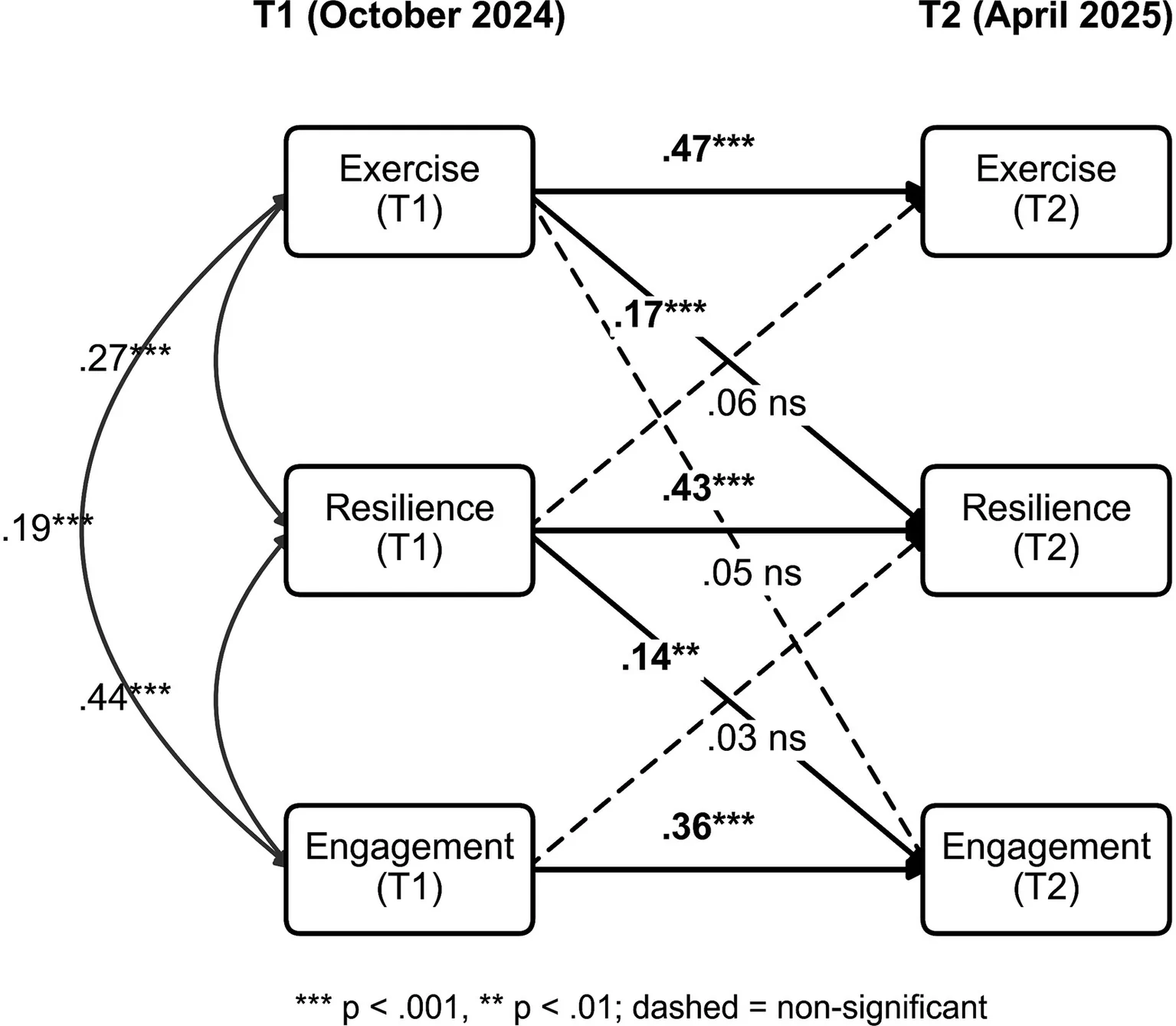
Standardized estimates for the cross-lagged panel model (*N* = 876). Solid paths are significant at *p* < 0.05; dashed paths are non-significant. T1 variables were freely correlated, and T2 residual covariances are omitted for clarity. *p < 0.05, **p < 0.01, ***p < 0.001.

### 3.4 Random-intercept cross-lagged panel model

The RI-CLPM also fit the data well (chi-square = 158.93, df = 106, *p* = 0.001; CFI = 0.979, TLI = 0.972, RMSEA = 0.036, 90% CI [0.025, 0.047], SRMR = 0.036), a Satorra-Bentler difference test indicated that it fit significantly better than the CLPM (delta chi-square = 30.69, delta df = 6, *p* < 0.001), and full parameter estimates appear in Table 3 and Figure 3. Under the identification constraints described above, the implied variance decomposition suggested that stable between-person differences accounted for approximately 43%, 37%, and 31% of the total variance in exercise, resilience, and engagement, respectively; these shares are model-implied quantities rather than directly estimable ones. At the within-person level, autoregressive effects remained significant for exercise (beta = 0.27, *p* = 0.005), resilience (beta = 0.22, *p* = 0.027), and engagement (beta = 0.16, *p* = 0.041). Consistent with Hypothesis 4, the within-person cross-lagged path from exercise to resilience remained significant (beta = 0.11, 95% CI [0.02, 0.20], *p* = 0.009): students who exercised more than their own average at T1 tended to report resilience above their own average at T2. In line with Hypothesis 5, the within-person path from resilience to engagement was attenuated and not significant (beta = 0.07, 95% CI [−0.01, 0.15], *p* = 0.074), suggesting that the engagement advantage of resilient students is chiefly a stable between-person difference rather than a short-term within-person process. Neither within-person reverse path was significant (resilience to exercise, beta = 0.04, *p* = 0.265; engagement to resilience, beta = 0.02, *p* = 0.583). Sensitivity analyses that fixed the PARS-3 indicator reliability to 0.70, 0.80, and 0.90 left the focal conclusion intact: the within-person exercise-to-resilience path was 0.10 (*p* = 0.014), 0.11 (*p* = 0.009), and 0.12 (*p* = 0.006) under the three assumptions, respectively, and the resilience-to-engagement path remained non-significant throughout. Within-person R-squared values were 0.08 for exercise, 0.07 for resilience, and 0.03 for engagement, so the modest magnitudes typical of within-person dynamics apply here as well.

**Figure 3:**
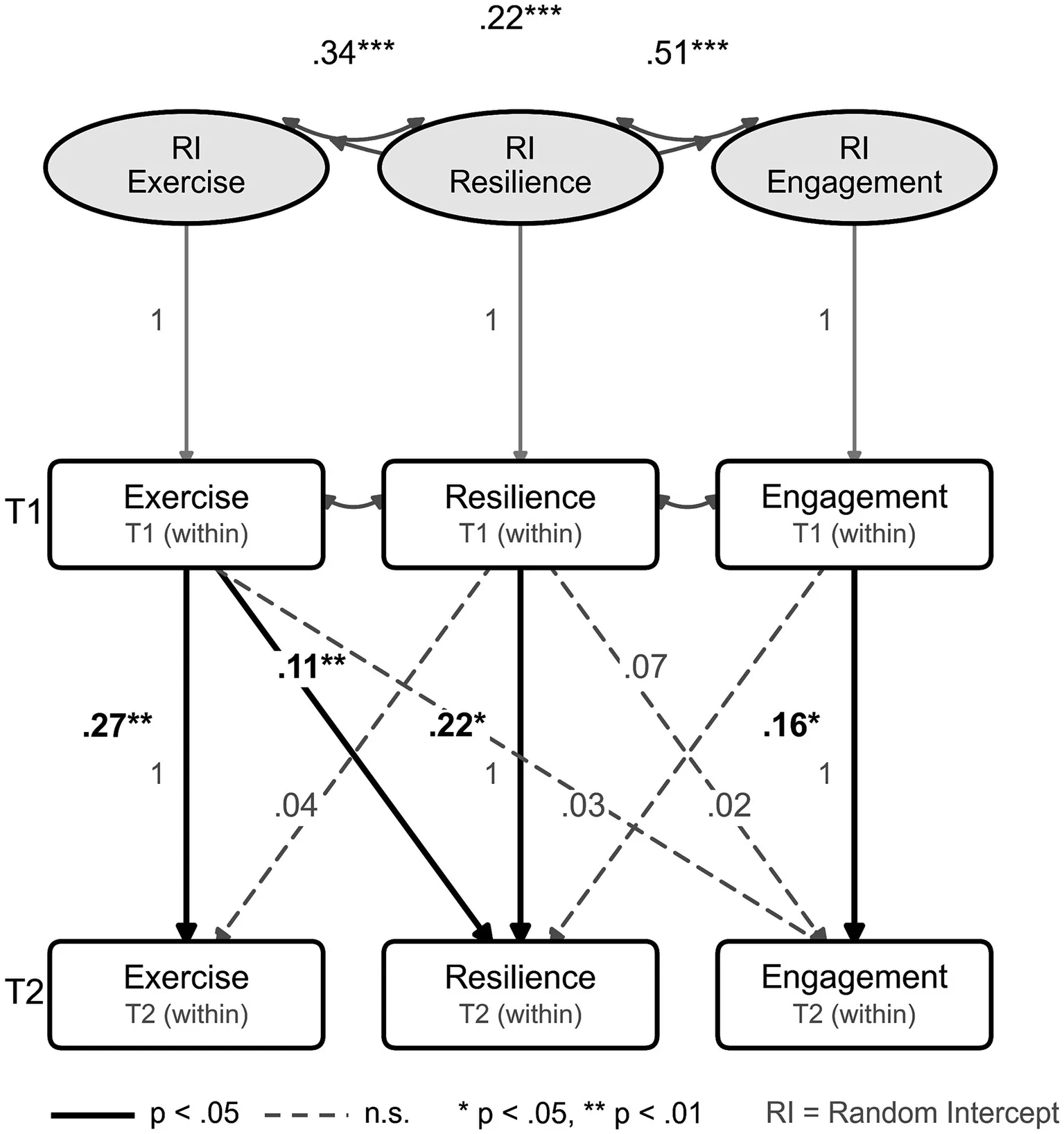
Standardized estimates for the random-intercept cross-lagged panel model (*N* = 876). Random intercepts carried loadings fixed at 1 on both occasions; the paths shown among the within-person components describe deviations from each student’s own baseline. Solid paths are significant at *p* < 0.05; dashed paths are non-significant. *p < 0.05, **p < 0.01, ***p < 0.001.

### 3.5 Moderation analyses

Multi-group analysis by gender revealed no significant moderation of the cross-lagged paths: constraining the paths to equality across men and women did not worsen fit (delta chi-square = 4.83, delta df = 4, *p* = 0.305). Age, entered as a continuous moderator through latent interaction terms, moderated neither the exercise-resilience path (beta = 0.04, *p* = 0.437) nor the resilience-engagement path (beta = −0.03, *p* = 0.559). The prospective associations identified above therefore did not differ by gender or age within this sample.

## 4 Discussion

### 4.1 Principal findings

This two-wave study followed 876 Chinese university students across a 6-month interval and estimated both a CLPM and an RI-CLPM for exercise volume, psychological resilience, and academic engagement. Three findings stand out. Students who reported more exercise at baseline reported higher resilience at follow-up, and this prospective association survived the separation of between-person variance as a within-person association under the identification constraints of the two-wave model. The resilience-to-engagement association, by contrast, behaved as a stable between-person difference rather than a short-term process. The direct exercise-to-engagement path was not significant in either model, offering no support for the time-displacement view but also no evidence of a direct resource transfer to engagement. Reverse paths and moderation by gender and age were null throughout.

### 4.2 Exercise volume and psychological resilience

The within-person exercise-to-resilience path (beta = 0.11) aligns with prospective cohort evidence on the clinical side of the same coin: in the HUNT cohort, regular exercise at baseline predicted a substantially lower risk of developing depression across follow-up even after adjustment for baseline symptoms and demographics (Harvey et al., 2018). Our estimate extends that pattern from clinical outcomes to a positive adaptation construct and from between-person comparison to within-person change. It also converges with two-wave cross-lagged evidence that physical activity predicts later resilience, and not the reverse, in Chinese adolescents (Guo and Liang, 2023). The mechanisms on offer fit this reading: manageable physical challenge operates as stress inoculation, fitness training recalibrates stress-regulatory physiology, and activity-generated positive affect builds durable resources (Silverman and Deuster, 2014; Fredrickson, 2001; Tugade and Fredrickson, 2004). None of these mechanisms requires the student to be an athlete; they require regularity, which is exactly what the PARS-3 volume score captures.

The magnitude deserves plain language. A one-standard-deviation increase in exercise volume relative to a student’s own baseline was associated with resilience higher by roughly 0.11 standard deviations 6 months later. That is a small association for any single student, and the present data cannot show whether it compounds across repeated semesters, whether students with the lowest baseline volume benefit differentially, or which forms of exercise provision work best; the study did not manipulate exercise, follow students beyond two occasions, or compare exercise modalities. These remain hypotheses for experimental and multi-wave investigation rather than implications of the current findings.

### 4.3 Resilience and academic engagement

The RI-CLPM placed the resilience-engagement link firmly between persons: resilience distinguished engaged students from disengaged ones, but short-term deviations in a student’s resilience did not forecast short-term deviations in engagement. This pattern helps reconcile two facts that sit uneasily together: cross-sectional studies consistently report sizable resilience-engagement correlations, whereas resilience-training programs produce small and inconsistent average gains (Joyce et al., 2018). If the correlation is carried mostly by stable characteristics, brief interventions should not be expected to move engagement much, and longitudinal work on school burnout and engagement likewise emphasizes slowly-moving trajectories rather than rapid shifts (Salmela-Aro and Upadyaya, 2014). The CLPM estimate converges with three-wave evidence that resilience prospectively predicts academic engagement in junior-secondary students (Bu et al., 2026); what the RI-CLPM adds is that, among university students, this association behaves as a stable between-person difference rather than a within-person process.

Two readings of this null are possible. Engagement may behave as a resource caravan in the COR sense: built slowly through sustained identification with a program of study, and insensitive to month-scale fluctuation in resilience (Hobfoll et al., 2018). Alternatively, the 6-month lag may simply be the wrong interval for a genuine but faster within-person process. Denser measurement would separate these readings, a point we return to below; emerging three-wave work in Chinese adolescents already treats resilience as a mediator of classroom effects on engagement (Gao et al., 2025), a design that our two waves could not support.

### 4.4 Reverse paths and moderators

Neither reverse path approached significance in either model: resilient students did not report more exercise later, and engaged students did not become more resilient, at least over 6 months. The selection effects often posited as alternatives to resource-building accounts were therefore not in evidence here. The null moderation results indicate that the observed associations did not differ by gender or across the narrow age band of undergraduate study in this sample. Because other potentially relevant moderators were not examined, these results should not be read as evidence about for whom exercise is most strongly related to resilience; that question requires designs that sample a wider range of participants and activity profiles.

### 4.5 Theoretical implications

The divergence between the CLPM and RI-CLPM estimates for the resilience-engagement pathway illustrates a general interpretive hazard. Had we reported only the CLPM, both cross-lagged paths would have invited a within-person reading, and the practical conclusion would have overstated the malleability of engagement through short-term resilience enhancement (Berry and Willoughby, 2017; Hamaker et al., 2015; Lucas, 2023). That the RI-CLPM fit better than the CLPM mirrors multi-sample comparisons showing superior fit for the RI-CLPM while the two models answer conceptually distinct questions (Orth et al., 2021; Mulder and Hamaker, 2021). Three levels of statement should be kept apart in this comparison. What is directly observed are the longitudinal zero-order associations and the CLPM estimates. What is estimated under the RI-CLPM assumptions and constraints are the between- and within-person variance components and the within-person paths, which are identified only through the constraints described in the Analytical strategy. What remains theoretical interpretation is any causal account of those paths: the better statistical fit of the RI-CLPM indicates that its decomposition is more consistent with the data, not that its substantive reading is necessarily the correct causal explanation. For the Study Demands-Resources framework, the results refine the resource metaphor: some resources, such as resilience built through exercise, behave dynamically over months, whereas engagement may require longer accumulation before measurable change occurs (Bakker and Demerouti, 2017; Hobfoll, 2002). A caveat accompanies these structural quantities: the variance decomposition is model-implied under the identification constraints required by a two-wave design and a single-indicator measure, not a directly estimable empirical quantity.

### 4.6 Strengths and limitations

The study has several strengths: a longitudinal design with a reasonably large sample, validated Chinese-language instruments, formally established measurement invariance, and the complementary use of CLPM and RI-CLPM with sensitivity checks on measurement reliability. Limitations qualify the conclusions. First, and most fundamentally for interpretation, the design provides only two measurement occasions and a single-indicator exercise measure. The RI-CLPM is therefore identified only through constraints on the exercise component variances and the indicator error variance, and the resulting variance decomposition and within-person paths are model-implied quantities rather than directly estimable components of the data. What these estimates support are temporally ordered associations under stated assumptions; they cannot trace full mediation chains, non-linear trajectories, or individual developmental processes, and they do not establish causal within-person effects (Maxwell and Cole, 2007; Mulder and Hamaker, 2021). The exploratory indirect estimate reported here is descriptive, and three or more waves would permit genuine longitudinal mediation tests and less constrained identification. Second, all measures were self-reported. Temporal separation, varied response anchors, and embedded attention checks reduce the scope for common method variance (Podsakoff et al., 2003), but the risk is not eliminated, and questionnaire-assessed exercise correlates imperfectly with device-measured activity (Prince et al., 2008). Third, the PARS-3 quantifies exercise volume and does not record modality, so the modality-specific mechanisms favored in the biological literature cannot be isolated with these data; the estimates should be generalized as volume effects. Fourth, the two waves fell in different semesters and seasons (October and April), so seasonal variation in exercise volume and study demands cannot be fully separated from within-person change. Fifth, unmeasured confounds remain: baseline wellbeing and positive affect could plausibly drive both exercise participation and later resilience, and regression to the mean offers an alternative reading of within-person estimates, one that the random-intercept structure partially but not fully mitigates (Bland and Altman, 1994). Sixth, the convenience sample came from universities in a single Chinese city, which limits geographic generalizability. Finally, the cross-lagged effects were modest in size, as is typical for within-person estimates, and should be read as average tendencies rather than deterministic rules.

### 4.7 Implications for practice and research

Within these limits, the findings are relevant to practice mainly as prioritization evidence. The prospective within-person association between exercise volume and resilience is consistent with the possibility that attainable increases relative to a student’s own baseline matter more than absolute performance standards; whether individualized progression, targeted provision for students with low baseline activity, or specific forms of exercise provision such as structured prescription are effective are questions for experimental evaluation, not conclusions of the present study. At most, the results support considering regular exercise programming as a candidate component of student mental-health support, pending experimental verification. For engagement-focused interventions, the results counsel patience: the resilience-engagement link behaved as a stable between-person difference, so gains in engagement may respond better to slower-acting levers such as curriculum design and academic identity than to short-term resilience enhancement. On the research side, priorities include three-wave and intensive longitudinal designs, experimental manipulation of exercise dose to test within-person causality, systematic recording of exercise modality as a potential moderator, and extension to adolescents and working adults.

## 5 Conclusion

In Chinese university students, exercise volume showed a temporally ordered, within-person association with psychological resilience over a 6-month interval, whereas the association between resilience and academic engagement was predominantly a stable between-person characteristic. Estimating both the CLPM and the RI-CLPM proved essential to this reading, and the central estimate was unaffected by the assumptions required for the single-indicator exercise measure; at the same time, with only two waves the within-person decomposition remains model-implied, and the findings are temporally ordered associations rather than confirmed causal effects. Promoting regular exercise therefore remains a promising candidate strategy for resilience support in university populations, pending experimental confirmation at the within-person level; claims about its direct association with academic engagement should be made more cautiously than is common in institutional advocacy.

## Data Availability

The raw data supporting the conclusions of this article will be made available by the authors, without undue reservation.
